# The Role of Electrocardiography in Occupational Medicine, from Einthoven’s Invention to the Digital Era of Wearable Devices

**DOI:** 10.3390/ijerph17144975

**Published:** 2020-07-10

**Authors:** Antonio Baldassarre, Nicola Mucci, Martina Padovan, Alessia Pellitteri, Silvia Viscera, Luigi Isaia Lecca, Raymond P. Galea, Giulio Arcangeli

**Affiliations:** 1 Department of Experimental and Clinical Medicine, University of Florence, 50134 Florence, Italy; nicola.mucci@unifi.it (N.M.); martina.padovan@unifi.it (M.P.); alessia.pellitteri@unifi.it (A.P.); silvia.viscera@unifi.it (S.V.); luigiisaia.lecca@unifi.it (L.I.L.); giulio.arcangeli@unifi.it (G.A.); 2 Postgraduate Training Programme, Mater Dei Hospital, MSD2090 Msida, Malta; raymond.p.galea@gov.mt; 3 Faculty of Medicine and Surgery, University of Malta, MSD2090 Msida, Malta

**Keywords:** electrocardiography, wearable electronic devices, occupational health and safety, health surveillance, health promotion, total worker health, Internet of things, biosensing techniques, medical informatics, history of medicine

## Abstract

Clinical-instrumental investigations, such as electrocardiography (ECG), represent a corollary of a procedures that, nowadays, is called upon as part of the principles of precision medicine. However when carrying out the professional routine examinations, most tend to ignore how a “simple” instrument can offer indispensable support in clinical practice, even in occupational medicine. The advent of the digital age, made of silicon and printed circuit boards, has allowed the miniaturization of the electronic components of these electro-medical devices. Finally, the adoption of patient wearables in medicine has been rapidly expanding worldwide for a number of years. This has been driven mainly by consumers’ demand to monitor their own health. With the ongoing research and development of new features capable of assessing and transmitting real-time biometric data, the impact of wearables on cardiovascular management has become inevitable. Despite the potential offered by this technology, as evident from the scientific literature, the application of these devices in the field of health and safety in the workplace is still limited. This may also be due to the lack of targeted scientific research. While offering great potential, it is very important to consider and evaluate ethical aspects related to the use of these smart devices, such as the management of the collected data relating to the physiological parameters and the location of the worker. This technology is to be considered as being aimed at monitoring the subject’s physiological parameters, and not at the diagnosis of any pathological condition, which should always be on charge of the medical specialist We conducted a review of the evolution of the role that electrophysiology plays as part of occupational health and safety management and on its possible future use, thanks to ongoing technological innovation.

## 1. Dawn of Electrocardiography

The first electrocardiograph for clinical purposes was developed by the Dutch physician Willem Einthoven, who was born in 1860 on the island of Java, at the time belonging to the Dutch East Indies. He received his medical degree and doctorate in 1885 in Utrecht, distinguishing himself by obtaining the Chair of Physiology at the University of Leiden just a year later. In 1924 he was awarded the Nobel Prize for Medicine for “the discovery of the electrocardiogram mechanism”.

The cardiac’s electrical nature was well known at the time, but there were no tools to study it. The study of electricity in the medical field, in fact, had already been started, about two centuries before Einthoven, by Gilbert (*De Magnete*, 1600), Bacone (*Novum Organum*, 1620) and Browne who, in the mid-seventeenth century, first used the term electricity (*Pseudodoxia Epidemica*, 1646).

William Gilbert, the personal physician of Elizabeth I, head of the College of Physicians and advocate of “magnetic philosophy”, introduced the term “electric”, derived from the Greek *ἤλεκτρον* (*elektron* = amber), to define the attraction of some light bodies obtained by the simple rubbing together of some objects. Gilbert described vis electrica, namely the resinous electricity, known today as static electricity, in his *De Magnete, magneticisique corporibus, et de magno magnete tellure*, published a few years before his death.

Thomas Browne, a physician, later called this attractive force as “Electricity, that is, a power to attract straws or light bodies and convert the freely positioned needle” in his masterpiece *Pesudodoxia Epidemica*, published in 1646. Among other things, Browne himself, while defining the task of the employees in the calculation of calendars, first introduced the term “computer”, namely “who computes”, referring to people who compute calendars.

Evidence of the electric nature in the animal kingdom moved forward in the following century. In 1769, the American Edward Bancroft suggested that the shock inflicted by the torpedoes was electric and non-mechanical, not as previously assumed by French scientist René-Antoine Ferchault de Réaumur in 1714 following what was initially hypothesized by the Italian physician Francesco Redi in 1671. Thus demonstrating similarities with the electric effect of Leyden’s jar, a device for storing electric charge that uses the same principle as modern capacitor (*An essay on the natural history of Guiana, in South America*, 1769).

At Bancroft’s suggestion, John Walsh, as witnessed in an epistolary correspondence with Benjamin Franklin, demonstrated a link between electricity and living beings (*Of the electric property of the torpedo*, 1773). This was practically at the same time as the studies of the Danish Peter Christian Abildgaard, published in *Tenramina electrica in animalibus instituta*, in 1775.

Joseph Priestley’s speculations regarding innate animal electricity aimed at locomotion. These were also supported by Felice Fontana in explaining the muscular motion, and were followed by the Italian Luigi Galvani, who demonstrated that direct contact with an electrical generator would lead to a muscle contraction in a frog’s leg. In a separate experiment, Galvani attached brass hooks, suspended from an iron railing in his garden, to the frog’s spinal cord, so as to observe how frog’s legs twitched during lightning storms. He, finally interpreted these results in terms of animal electricity, later known as galvanism, which is the basis of modern electrophysiology (*De Viribus Electricitatis in Motu Musculari commentarius*, 1791) Similar evidence was demonstrated by the Dutchman Swammerdam over a century earlier (*Bible of Nature*) in 1646. Galvani devised an instrument for measuring this animal electricity, the galvanometer, precisely named after its inventor that represent essentially what an electrocardiography (ECG) is: a sensitive galvanometer.

Few decades later, the astatic galvanometer allowed its inventor, Leopoldo Nobili, first (*Memories and Observations*, 1834), and Carlo Matteucci, later (*Traité des phénomènes électro-phisiologiques des animaux*, 1844), to measure and record the electrical activity in some Anurans specimens.

Matteucci, a contemporary of the German du Bois-Raymond (*Untersuchungen über thierische Elektrizität*, 1848), described the nervous action potential using a galvanometer. This was, subsequently amended by William Thompson, best known as Lord Kelvin, for applications in the field of telegraphy, inventor of the mirror galvanometer (1858) and siphon recorder (1867), which had presumably been used to perform the first electrocardiography at St Bartholomew’s Hospital in London in 1870 by Alexander Muirhead (Elizabeth Muirhead. Alexander Muirhead 1848–1920. Oxford, Blackwell: privately printed 1926).

**Figure 1:**
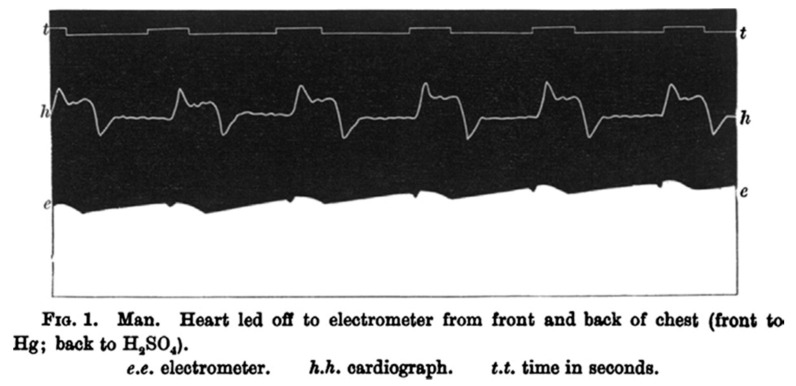
First human “electrogram”, performed by Waller, showing an electrical activity preceding every heartbeat.

At the end of the century, the British physiologist Augustus D. Waller, using a device for detecting small rushes of electric current, the capillary electrometer invented by Gabriel Lippmann earlier in 1873. He was able to record the cardiac electrical activity of a human being for the first time, defining this recording “electrogram” (*A demonstration on man of electromotive changes accompanying the heart beat*, 1887) as shown in Figure 1.

**Figure 2:**
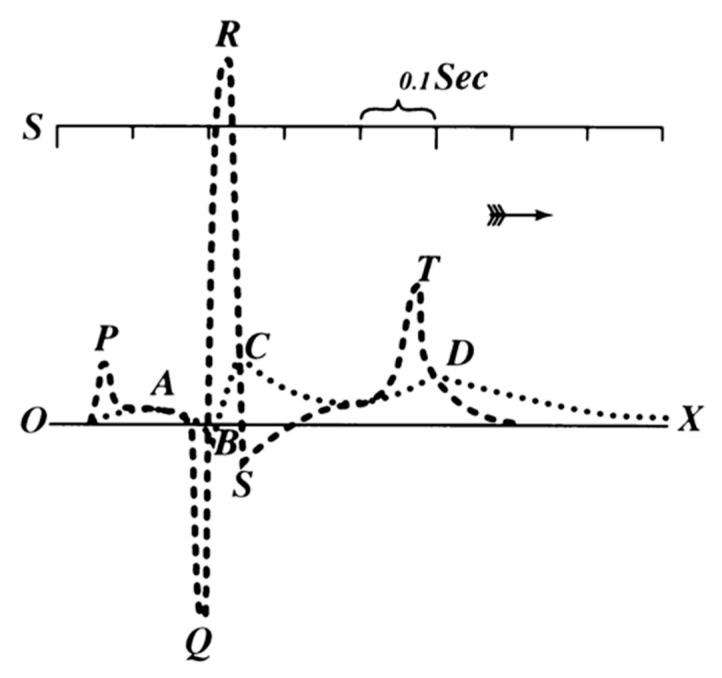
Einthoven superimposed two electrocardiographs (ECGs), in order to highlight the differences in the two tracks. The one labelled with ABCD was obtained with the string galvanometer while the one labelled with PQRST was obtained by Einthoven with the application of a mathematical correction.

Einthoven introduced the term “electrocardiogram” in 1893 and also improved the electrometer, introducing a correction formula that made it possible to distinguish five deflections, which he named after the cartesian nomenclature PQRST [1,2] as shown in Figure 2.

**Figure 3:**
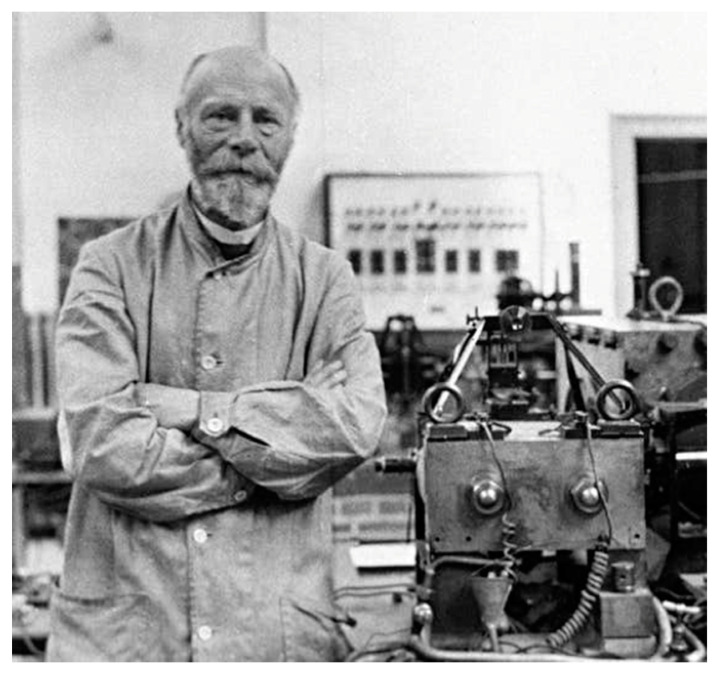
Willem Einthoven alongside his invention, the string galvanometer.

In 1901, Einthoven revealed to the scientific community the string galvanometer (Figure 3), using a fine quartz string coated in silver based instead of the wire coil conceived by Deprez and d’Arsonval, weighing about 300 kg, and on 22 March 1905, he managed to transmit via cable, 1500 m away from his laboratory, an ECG record, also putting into place the premises of telemedicine.

Following in Einthoven footsteps and, later, actually working with him, Thomas Lewis studied the heart excitatory mechanisms and rhythm disorders. He summarised his findings, in his work “Mechanism and Graphic Registration of the Heart Beat”, definitively moving the study of electrocardiography from the bench to the bedside. This was also recognized by Einthoven during his Nobel lecture [1,3].

Thomas Lewis’ student, the American physiologist Frank N. Wilson, laid the foundations of modern electrophysiology, first standardizing its methodologies in the mid-1900s [4].

In 1928, Frank Sanborn’s company converted their table model electrocardiogram machine into their first portable version, weighing about 25 kg, and powered by a 6-volt automobile battery [5].

A further step forward was made, in 1937, by the Japanese physician Taro Takemi, who introduced the first portable electrocardiograph machine [6].

One of the first attempts in terms of conjugation of clinical monitoring and portability was that attempted by the American physician Norman Jeff Holter in 1949, who created a backpack, weighing about 35kg, containing the instrument capable of recording the cardiac activity of the wearer [7].

A continuous improvement, over the years, in terms of usability, going from five operators to one single operator necessary to record the ECG trace, and portability, from about 300 kg to about 1 kg, has been made possible by the progress of science and technique that was achieved.

Not all technological advances, however, have resulted in clinical practice, such as, for example, the one introduced by Baule and McFee in 1963, the first to detect the magnetocardiogram, the electromagnetic field produced by the heart electrical activity [8]. Their method could detect the ECG without the use of skin electrode patches but, although a potentially useful technique, it has never gained clinical acceptance, because of its cost.

Following the work of Einthoven, Lewis and Wilson, the use of commercial ECG machines has become increasingly common and remains, to date, one of the most useful clinical-instrumental devices used in medical practice, even for the occupational physician. The advent of the digital age, of silicon and printed circuits, has permitted miniaturization of the components making up the devices, including the electromedical ones. Since some years now, the adoption of wearables in medicine has rapidly expanded worldwide.This wasmainly driven by consumers’ demand to monitor their own health. With the ongoing research and development of new features capable of assessing and transmitting real-time biometric data, the impact of wearables on cardiovascular management has become inevitable. Despite the potential offered by this technology, as evident from the scientific literature, the application of these devices in the field of health and safety in the workplace is still limited, which is also due to the lack of targeted scientific research.

## 2. Materials and Methods

Wearable devices, also known as “wearables”, are a category of electronic devices that can be worn as accessories or embedded in clothing. The devices are hands-free gadgets with practical uses, powered by microprocessors and enhanced with the ability to send and receive data via the Internet.

The rapid adoption of such devices has placed wearable technology at the forefront of the Internet of things (IoT). The past few years have seen rapid development and introduction of wearable technology products adapted for medical and healthcare uses.

We proposed a narrative review of the literature, which was carried out in order to provide a broader search of the existing literature and a comprehensive description of a given theme, allowing for the identification of gaps in scientific knowledge.

The P.I.C.O. strategy was designed as follows [9]:

- Population: *workers*;
- Interest: *cardiovascular monitorization*;
- Context: *occupational medicine*;
- Outcome: *possible use in occupational safety and health (OSH)*

**Figure 4:**
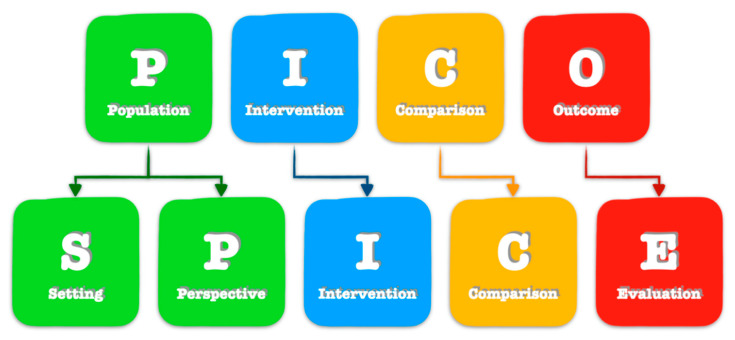
P.I.C.O./S.P.I.C.E. research strategy.

We also ran a S.P.I.C.E. strategy approach, built upon P.I.C.O., as illustrated in Figure 4. First, the population component is split into two parts: setting and perspective. Second, outcomes is replaced with evaluation, in order to encourage a broader evaluation framework and incorporate concepts such as “outputs” and “impact” together [10].

The S.P.I.C.E. strategy was designed as follows:

- Setting: *occupational settings*;
- Perspective: *worker*;
- Intervention: *cardiovascular monitorization with smart wearable devices*;
- Comparison: *cardiovascular monitorization with ambulatory instruments*;
- Evaluation: *possible use in occupational safety and health (OSH)*

The research strategy, based on PubMed, was defined as follows:

- Ad hoc string: (ECG OR electrocardiograph* OR electrocardiogram* OR EKG OR cardiogram*) AND ((wearable* OR mobile* OR portable*) AND (device* OR sensor* OR technology*))
- Article type: reviews and systematic reviews
- Publication date: last 5 years (since 2015)
- Language: English

We also tried to run a research on EMBASE as follow:

- Ad hoc string: *(‘worker’/exp OR ‘laborer’ OR ‘labourer’ OR ‘worker’) AND (‘cardiovascular monitoring device’/exp OR ‘wearable device’ OR ‘smart device’) AND ‘review’/it*

**Figure 5:**
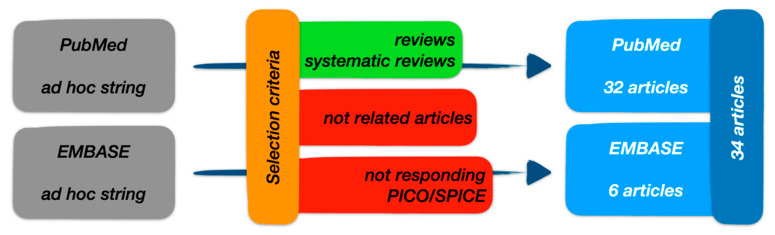
Research strategy flow-chart.

We obtained 79 search results from PubMed and 157 search results from EMBASE, respectively. We included articles with an abstract or full document available online, even if they were not in open access, published in the period between 2015 and 2020. We excluded articles not related to the theme as well as those that did not answer the research question as identified in the P.I.C.O. criteria. After applying the exclusion criteria and duplicates removal, 34 articles were finally selected from PubMed and EMBASE search results pools, as reported in Figure 5. The survey was conducted in April 2020.

## 3. Results

The 34 articles that met the P.I.C.O./S.P.I.C.E. criteria adopted for this narrative review are summarized in the Table 1.

## 4. Evolution of Electrocardiography and Future Perspective

### 4.1. Wearable Smart Device Architecture

Over the last twenty years, we have witnessed an increase of the spread and the pervasiveness of wearable devices, mainly due to the advancement of microelectronics, which allowed for smaller and more power efficient cost-effective integrated circuits, as well as, the invention of micron sized sensors, which can be integrated directly inside the silicon die of the integrated circuit. Biomedical sensors have been integrated directly with the signal processing and radio transmission electronics, and we can currently see an explosion of off-the-shelf wearable devices for biological sensing and monitoring. An important concept in wearable devices architecture is the Body Area Network (BAN): a cluster of devices built around a host (usually the user’s smartphone), and interconnected generally thought a wireless communication channel, such as Wi-Fi or Bluetooth. The sensor itself is only the first part of the signal processing chain: the raw data need to be extracted and processed before it can be useful to a data logger unit (DLU). This system is in general very simple, small, cost-effective and of low power, because its first requirement is to be non-invasive and with a long battery life. As a result of this, DLUs have a very limited computational power, demanding that the data elaboration tasks are assigned to the central unit of the BAN. Depending on the application, acquired sensor data can be streamed to a remote device/server for offline monitoring, or it can be used in real-time when is necessary to provide immediate feedback to the user [11].

Conventional rigid sensors, with which the devices on the market are currently equipped, are normally silicon-based, causing some limitations in terms of flexibility, a fundamental feature for the ergonomics of these wearable devices. As a result of which, graphene-based sensors researches have boomed in recent years, aiming to replace silicon sensors for both non-invasive flexible wearable sensors as well as invasive devices. Graphene has many advantages in mechanical, thermal and electrical properties, with respect to classical microelectronics semiconductors. For example, it has excellent electrical conductivity, high optical transmittance, superior thermal conductivity and outstanding mechanical flexibility. Despite these premises, the graphene technology currently has some limitations in terms of durability and safety, requiring further development before possible commercial use [12].

Wearable electronics are also often embedded into clothing in order to sense, measure, monitor and report human vital signals, for example, heart muscle signals that can be recorded by ECG or electromyography (EMG) garments, respectively. One example of wearable ECG is NUUBO®, worn like a sports bra with electrodes positioned tightly but comfortably in the right locations to get high quality data. Unlike conventional gelled electrodes, textile electrodes used in ECG garments are more comfortable and user-friendly, especially for the continuous monitoring of cardiac conditions. The quality of textile-based ECG electrodes is dependent on different factors, such as skin-textile contact, humidity, contact pressure, electrode placement, user’s movements and muscle activity. Regarding placement, textile electrodes that are placed on areas with fewer muscles could provide better quality data. The optimal positions of the electrodes considering the body movements have been measured by different methods, such as a robotic running mannequin and 3D body scanning [13,14].

### 4.2. Monitoring of Cardiac Function in the Era of Wearable Devices

In 2011, the World Health Organization affirmed that “the use of mobile and wireless technologies to support the achievement of health objectives (mHealth) has the potential to transform the face of health service delivery across the globe.” As an example, the use of short message service (SMS) and mobile phone apps represent two simple but effective prevention strategies for cardiovascular diseases. Text messaging has been shown to be effective for helping and goading people in terms of weight loss, physical activity and in the management of blood pressure and diabetes. Smartphone apps, are also used, for example, to program home-based cardiac rehabilitation. mHealth has the capacity to reduce costs and control the burden of cardiovascular diseases [15].

Focusing on management of cardiovascular disease risk, five studies were considered. The Treatment of cardiovascular Risk in Primary care using Electronic Decision support (TORPEDO) study demonstrated that a computer-guided intervention is a tool for screening for risk factors for cardiovascular disease (CVD) in primary care. The COnsumer NavigatioN of Electronic Cardiovascular Tools (CONNECT) study tested a consumer-directed responsive web-app. The Tobacco, EXercise and diet Messages (TEXTME) study used text messaging to provide motivation and information about diet, physical activity and encourage smoking cessation. The mobile application FOODSWITCH allows consumers to scan the barcodes and have information about the nutritional characteristics of packaged foods. Finally, SEARCH AF (screening for atrial fibrillation (AF) using an iPhone electrocardiogram (ECG) in pharmacies), which utilised smartphone hardware to provide widespread screening for AF using a real-time single lead ECG assessment [16].

Portable, out-of-hospital, electrocardiography (ECG) is an important medical sensor technologies innovation. There is a heterogeneity in terms of available devices, as they can be classified as single limb lead ECG and multiple-lead devices. Devices like AliveCor® KardiaMobile, Omron™ Heart-Scan typically belong to the first category and use the touch sensation of fingers, thumb, wrist or palm to capture electrical signals. On the other hand, multiple-lead devices like iRhythm™ Zio® patch could capture two or more ECG leads used sensors placed on the chest wall. Single-lead devices have a capability to detect abnormal rhythms, while multiple-lead devices use chest patch/electrodes and may have a utility to detect both abnormal rhythms and localization of ischemic abnormalities [17,18].

In the last few years, some wireless ECG monitoring systems have been developed using flexible and dry capacitive electrodes for long-term monitoring of cardiovascular health. Conversely, capacitive-coupled dry electrodes can measure ECG signals over a textile-based interface material between the skin and electrodes such as cotton, which—in comparison with other materials, such as wool, silk, or nylon—has a higher dielectric constant. Moreover, the proposed system does not require a ground electrode, and ECG can be measured by using only two smaller electrodes for better portability and convenience for the user. The proposed ECG electrodes can run continuously for 2 weeks and for long-term monitoring, acquiring the ECG signals with the QRS complexes, P and T waves clearly distinguishable. The electrodes are connected to a data acquisition system that receives the raw ECG signals and transmits the data to a computer using Bluetooth. A software application also has been developed to process, store and display the ECG signal in real time [19].

The choice between the various technologies also depends, in fact, on the duration of the required monitoring period taking into consideration the symptoms and previous pathologies. It also depends on the subject ability to initiate ECG recording and the tolerability of the device as well as the environmental conditions in which it is to be used [20].

**Figure 6:**
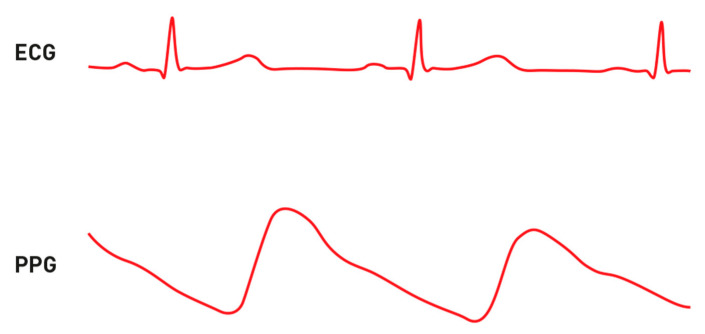
1-lead ECG and photoplethysmography (PPG) example traces.

The technology behind these devices consists of sensors, algorithms and electrodes that allow cardiovascular monitoring, such as measurement of heart rate variability (HRV) via photoplethysmography (PPG) and ECG (Figure 6). HRV reflects the combined activity of sympathetic and parasympathetic tone on the heart rate. It is defined as the physiological variation in the duration of intervals between sinus beats and serves as a measurable indicator of cardiovascular integrity and prognosis. The major methods of analysis can be divided into time-domain that assess the difference between normal R-R intervals, excluding ectopic beats, and frequency-domain, allowing for the distinction between high frequency (HF) and low frequency (LF) components [21].

It is well documented that PPG, works essentially thanks to the blood absorbance of the green wavelength of the visible spectrum and the reflectance of the infrared respectively. The measurement is performed by using a high frequency (AC) pulsation, which can capture the heart beat variations and a baseline light (DC), which is used to remove the artefacts due to respiration, the sympathetic activity and thermoregulation. Therefore, using green LEDs and associating them with photodiodes, or infrared sensors, a device is able to detect the amount of blood flow, thus, measuring the heart rate (HR) in beats per minute (BPM) and evaluate its variability (HRV). The principal difference between PPG and conventional ECG is in the type of signal captured. ECG analyses heart electrical activity, while PPG measures the propagation of the peripheral pulse (PP) wave. A fundamental parameter is the pulse transit time (PTT), which represents the time of propagation of the PP wave from the heart to the distal arterioles and measure the time that spend between the R-wave of QRS complex in the ECG and the arrival point in PPG. Several studies have shown how PTT represents a marker of the autonomic nervous system in parallel with HRV. PTT is also negatively correlated with age, blood pressure and arterial stiffness [22].

Innovations in big data analysis with machine learning has culminated in the development of deep neural networks to identify patients with AF based on PPG guided R–R variability alone. Furthermore, the latest iteration of wearables now uses a hybrid system that prompts the user to acquire a single-lead ECG, when their HR deviates from a personalized R–R variability and physical activity template generated from their PPG data. These systems are likely to compete with conventional medical grade devices, given the ease with which biometric indices can be recorded [23].

A meta-analysis study evaluated smartphone apps to measure heart rate compared with a validated method. In adults, the result showed no significant difference. The Pearson correlation coefficient of the relation between heart rate measurement with a smartphone and a validated method was always ≥0.90. These results suggest that a smartphone app deriving heart rate from a PPG signal could be used as an alternative for already validated methods, such as an ECG or pulse oximeter in an adult population in resting sinus rhythm, for heart rate measurements between 60 and 100 beats per minute [24].

Smartphones and smartwatches have become increasingly powerful, mostly thanks to electronic circuits miniaturization and to the strengthening of the Wi-Fi network. New electronic devices can also monitor heart rhythm in a device that can easily be worn. Smart watches generate a 30 s intermittently single lead electrocardiogram that can be displayed on the phone or watch screen. That signal can reveal arrhythmias like atrial fibrillation (AF), capable of activating integrated alarms that can warn the user or previously selected third parties. By directly selling to consumers, wearable devices can increase the number of people who are monitored during their daily activities, also serving as an excellent resource for continuous ECG monitoring with data saved in the cloud, which can then be readily assessed from anywhere. Other devices, like Holter monitors, are limited by the intermittent nature of monitoring and the need to be returned for analysis. New devices represent a non-invasive method for prolonged periods of screening compared to the invasive implantable loop recorders [25]. Single-lead portable ECG devices, moreover, may offer an efficient screening option for AF compared with 24 h Holter monitoring. Total monitoring time is related to AF detection, and a total of 19 min may achieve a similar detection rate to 24 h Holter monitoring [25].

In fact, they can accurately discriminate AF from sinus rhythm with more than 93% sensitivity and 84% specificity, compared with the ECG. Wearable devices can also help in the prevention of cryptogenic stroke as reported in the 30-Day Cardiac Event Monitor Belt for Recording Atrial Fibrillation after a Cerebral Ischemic Event (EMBRACE) trial [26,27,28,29,30].

In contrast to other available AF detection algorithms, the Apple Watch algorithm has been the first to receive Food and Drug Administration (FDA) clearance for the consumer market. Rhythm analysis is reported after 30 s of recording and is best done at rest. Rhythm classification as AF or sinus is only achievable reliably during rest, due to significant noise artefacts with arm movement. The app classifies an iECG as sinus rhythm (SR), AF or inconclusive. Recordings from the watch are saved in PDF format in the Health native built-in app. iECG advantages include possibility of AF screening in high risk population and direct access to personalized health measures, such as intermittent anticoagulation. Moreover, for patients taking antiarrhythmic therapy with a “pill in the pocket” method prompt rhythm identification via the Apple ECG application could be helpful. However, this ECG algorithm performance is limited in classifying other arrhythmias like second or third-degree AV block, bigeminy, frequent ectopy and junctional rhythm [31,32].

This technology is also used for mindfulness applications, which focus the user on relaxation, as well as providing information on the average BPM during walking, resting and heart rate variability. To overcome artefacts due to motion and skin characteristic, many devices integrate inertial sensors, such as accelerometers and gyroscopes into the data processing pipeline, in a technique called sensor fusion. While many studies have established a high correlation between PPG and ECG for HRV at rest, few studies have assessed HRV parameters with exercise. Susceptibility to noise is demonstrated by HF coherence decrease after exercise. Several studies show how HRV is predictive of all cardiovascular morbidity using clinical ECG recordings studied at rest, with exercise and in the ambulatory setting. Hillebrand and colleagues demonstrated the protective effect of elevated HRV and the association between lower HRV and an increased risk of first CV event in patients without known CV disease. For these reasons, over recent years HRV has increasingly been used to monitor the status of athletes. Exercise-induced HRV variables provides valuable prognostic information that can contribute to establish cardiovascular risk scores. Negative exercise performance outcomes are generally associated with HRV reduction [33,34,35,36].

New devices could improve diagnostic precision and facilitate collaboration between physicians, enabling also the possibility of virtual consultation. Ultrasound measurements can be executed with a simple phased-array probe connected directly to a smartphone, where data are sent and stored via cloud-based apps. Digital stethoscopes currently can record and share heart sounds phonograms via their accompanying app. Additionally, in medication management area, new stick-on devices coupled with sensor-enabled pills could better track medication adherence [37].

Advances in wearable tracker technology can also facilitate the care of the aging population, and can have a significant impact on countries’ healthcare systems. These devices can encourage users to increase their daily activity and to decrease their waist circumference, they also may facilitate AF diagnoses and help in predicting hospital length of stay. Unfortunately, most related researches are derived from studies on children and young adults, with a lack of studies focusing on elderly people. Moreover, there is no standardized method for quantifying data from wearable devices across multiple studies [38].

Wearable technologies can produce the greatest impact in cardiovascular prevention. Studies have demonstrated a reduction in office visits and an increase in patient satisfaction. However, there are still important limitations that limit the adoption of wearable technologies in medical practice, such as cost, the uncertain clinical implications and high sensitivity, which may lead to the over detection of benign no clinically relevant dysrhythmias. Their use can lead in spurious diagnoses, unnecessary investigation and patient anxiety [39].

Smart wearable devices have the potential to change the ways to collect and process health data. Technology application spectrum indeed is very wide. As seen, cardiovascular diagnostic area is the major focus, but their use in the diagnosis and monitoring of neurologic, metabolic and rheumatology diseases is increasing [40,41].

### 4.3. Wearable Devices: Potential and Criticality in the Working Context

Several studies assessed if wearable devices such as Fitbit activity tracker or mobile app installed really provide a benefit for health outcomes. Wearable devices collect behavioural data on an ongoing daily basis so that it allows health care providers to monitor patients and to provide personalized intervention. In addition, more employers and insurers are likely to offer wearable devices as part of a wellness program [41,42].

Wearable technology, increasingly widespread in the infotainment market, is starting to play a crucial role in protecting the health and safety of workers, allowing the monitoring of vital parameters while signalling emergency situations, such as rhythm alterations. The wearable devices provide the user with services and information, whilst continuing to run other activities in the background, often taking the place of a personal trainer by encouraging to make better decisions through monitoring of the physiological and behavioural parameters. Devices that combine the measurement of physiological parameters, such as the heart rate, data analysis and machine learning functions, are also defined as “physiolytic”, and they offer an increasingly personalized and customized user experience, going beyond simple traditional measurements. Other features, such as localization, detection of fall, tripping and slipping, are valid solutions in particular situations, such as solitary workers working in the railway networks.

Also, with the localization functionality, mainly based on Global Positioning System (GPS) technology, these devices have the potential to help companies redesign spaces and reorganize work so as to improve the productivity, as well as facilitating eventual rescue operations in case of emergency (ICE). Such devices could, for example, help a transport company to determine how to position workers more efficiently on a loading platform.

In the literature, the adoption and implementation of these devices in corporate wellness programs has already been reported, so as to improve health and safety, in some work situations [43,44].

The use of such devices in a work environment, however, is indeed not without potential problems, due to the massive amount of data collected, which companies could exploit and reuse for other organizational objectives, such as redundancy policies or other drastic changes, which could involve the same workers. Any smart device owner, in fact, is an unconscious “data generator” that provides, for free, to technological giants, companies and institutions the opportunity to exploit big data, estimated by the exabyte, namely 1018 bytes. In the above-mentioned context, current as future, risks deriving from the insecurity and unpredictable behaviour of employees should be assessed, if they perceive this technology as a threat and not an opportunity.

Furthermore the use of AI, in wearable ECG monitors, also raises potential legal and ethical concerns. Though it can power highly accurate algorithms, AI is a “black box” that is difficult to interrogate if it gives an unexpected output. AI is also susceptible to racial, gender and socioeconomic bias in training datasets, potentially perpetuating harm to underserved groups. The challenges faced by AI are not unique to wearable ECG monitors, but will need to be overcome if the full potential of AI in medicine is to be realized. Security and privacy are two other related domains in which wearable ECG monitors will be tested. Wearable ECG monitors contain some of an individual’s most sensitive data [45].

Looking specifically at the European regulations, the Medical Device Directive was replaced by the new Medical Device Regulation. Although the new regulatory framework still revolves around intended purpose, the bar for medical device classification has been lowered, because of a broader definition assigned to the term medical purpose. In short, despite the undisputed potential of such apps in the detection of arrhythmias, adhering to appropriate laws and regulations remains a significant hurdle to address [45,46].

Machine learning can further increase the accuracy by extracting more features from various biosensors. Future technology-supported interventions for health and wellness will require the data collected from biosensors, to be integrated with other sources of information. Data, in fact, must be represented as meaningful information for health-related decision-making by a range of stakeholders, including patients, family members, health care providers, public health professionals and, last but not least, policy makers. The application of these devices in the field of occupational health and safety is far from a reality yet. The opportunities offered by this type of technology, therefore, are many and could have a positive impact on the health and safety process, and on the prevention of accidents and pathologies, especially in particularly risky working environments. They offer a new range of possibilities for targeted health promotion through, the reduction of sedentary behaviour at work and physical inactivity, thanks to parametric sensors and specific applications—native or third-party—installed on devices, suitable for performance monitoring, encouragement and moving goals to be achieved within 24 h, thus leading to healthy and good living habits.

The push of industry 4.0 has certainly confirmed a step forward. These devices, in fact, will be capable of exploiting Internet of things technology (IoT), especially on the eve of 5G technology, and also interfacing with electronic medical records on smartphones, project telemedicine towards new horizons and challenges, such as the better management of workers and the reduction of the relative direct and indirect costs. By leveraging IoT technologies, it is possible to transform almost any Personal Protective Equipment (PPE) into smart-PPE, able to handle simple to complex occupational accidents, and eventually to save lives. Technological giants, driven by the trend of continuous growth worldwide, are working on the addition of new functions, such as sleep monitoring and blood glucose measurement, allowing the users themselves a more complete collection of clinically relevant data and recording it on their own electronic health records.

**Table 1:** Research strategy selected articles.

N	Title	Year	First Author	Country	Method	Key Message	PubMed	EMBASE
1	Lead-I ECG for detecting atrial fibrillation in patients with an irregular pulse using single time point testing: a systematic review and economic evaluation [17]	2020	Duarte R	UK	Systematic review	Single time point lead-I ECG devices appear to be a cost-effective compared with manual pulse palpation (MPP) followed by a 12-lead ECG.	✓	
2	Wearing Your Heart on Your Sleeve: the Future of Cardiac Rhythm Monitoring [41]	2019	Al-Alusi MA	USA	Review	Wearable ECG monitors are currently most useful to detect atrial fibrillation. Further study is needed to demonstrate whether wearable ECG monitors improve patient outcomes, and to expand their use into other indications.	✓	✓
3	How useful is the smartwatch ECG? [30]	2019	Isakadze N	USA	Review	The Apple Watch Series 4 ECG feature is FDA cleared for detection of presence of AF. AF represents only one area where the Apple Watch ECG shows promise to transform care.	✓	
4	Apple Watch, Wearables, and Heart Rhythm: where do we stand? [25]	2019	Raja JM	USA	Review	Health monitoring devices can be used as a non-invasive, ambulatory assessment of heart rate and rhythm. These devices have even shown to be cost-effective when used in a community screening program.	✓	✓
5	Electrode placement in electrocardiography smart garments: A review [13]	2019	Soroudi A	Sweden	Review	Since a successful ECG monitoring garment needs a multi factor design, a good strategy would be a cooperative design using techniques and experiences provided by medical experts, textile and garment designers.	✓	
6	Mobile Self-Monitoring ECG Devices to Diagnose Arrhythmia that Coincide with Palpitations: A Scoping Review [46]	2019	Marston HR	UK	Review	Mobile heart monitoring devices has several benefits: alleviation of patient anxiety, lowering the risk of morbidity and mortality, while progressively influencing national and international care pathway guidelines.	✓	
7	The use of photoplethysmography for assessing hypertension [33]	2019	Elgendi M	Canada	Narrative review	High Blood Pressure (BP) is a major source of mortality and morbidity around the world., Most PPG-based BP estimation is mainly divided into two research directions based on waveform morphology theory and waveform propagation theory.	✓	
8	Is There a Benefit to Patients Using Wearable Devices Such as Fitbit or Health Apps on Mobiles? A Systematic Review [40]	2019	Jo A	USA	Systematic review	This systematic review found a limited benefit to the use of a wearable device in chronic disease management. Findings showed inconsistent conclusions with respect to significant improvements in weight loss, blood pressure, and cholesterol level, except HbA1c.	✓	
9	Ambulatory ECG monitoring in the age of smartphones [24]	2019	Sanders D	USA	Narrative review	Consumer-oriented wearable devices are aimed at arrhythmia monitoring, which could lead to increased arrhythmia detection, but at the risk of more false-positive results and excessive use of healthcare resources.	✓	
10	Graphene-Based Sensors for Human Health Monitoring [12]	2019	Huang H	China	Narrative review	As a novel 2D material, graphene has aroused a boom in the field of sensor research around the world due to its advantages in mechanical, thermal, and electrical properties. Numerous graphene-based sensors used for human health monitoring have been reported.	✓	
11	Wearable devices for cardiac arrhythmia detection: a new contender? [22]	2019	Sajeev JK	Australia	Review	Potential patient-specific barriers may impede widespread screening using SDP. However, attitudes to SDP-based arrhythmia detection remain favourable compared with conventional Holter monitoring system for symptomatic arrhythmia.	✓	
12	Data management and wearables in older adults: A systematic review [36]	2019	Alharbi M	Australia	Systematic review	Wearable trackers are generally valid, reliable, and/or feasible when tracking step counts, MVPA, ECG and HR in aging populations. Thus, trackers may be ideal to help in diagnosing, measuring, monitoring, and/or motivating in this population cohort. There needs to be a framework and/or guideline and a standardized method for the collection and analysis of wearable tracker data.	✓	
13	The Current State of Mobile Phone Apps for Monitoring Heart Rate, Heart Rate Variability, and Atrial Fibrillation: Narrative Review [45]	2019	Li KHC	China	Narrative review	There is a role for mobile phone app in the diagnosis, monitoring, and screening of arrhythmias and HR. Within the context of HR monitoring and AF detection, given the impressive degree of sensitivity (>90%) and specificity (>90%) in most cases or apps, neither sensitivity nor specificity is more important than the other.	✓	
14	The Accuracy of Acquiring Heart Rate Variability from Portable Devices: A Systematic Review and Meta-Analysis [27]	2019	Dobbs WC	Switzerland	Systematic Review and Meta-Analysis	HRV measurements acquired using portable devices demonstrate a small amount of absolute error when compared to ECG. Portable devices were influenced by metric, position, and biological sex. Practitioners and researchers should consider the cost–benefit along with the simplicity of the measurement when attempting to increase compliance in acquiring HRV measurements.	✓	
15	The Clinical Value of Heart Rate Monitoring Using an Apple Watch [29]	2019	Karmen CL	USA	Review	Recent reports of the series 4 Apple Watch show that these devices can accurately discriminate atrial fibrillation from sinus rhythm with more than 93% sensitivity and 84% specificity compared with the ECG.	✓	
16	A systematic review of noninvasive electrocardiogram monitoring devices for the evaluation of suspected cardiovascular syncope [42]	2019	Solbiati M	Italy	Systematic review	This is an area of medicine where precision medicine, managing patients based on their own individual needs with the most appropriate device, is important.		✓
17	Electronic textile electrocardiogram monitoring in cardiac patients: a scoping review protocol [14]	2018	Teferra MN	Australia	Systematic review	E-textiles are fabrics (or clothing) that contain electronic elements or circuits woven directly into the material and are an emerging interdisciplinary field of research.		✓
18	Heart Rate Variability: An Old Metric with New Meaning in the Era of using mHealth Technologies for Health and Exercise Training Guidance. Part One: Physiology and Methods [31]	2018	Singh N	USA	Clinical review	Recently, the availability of commercially available heart rate (HR) monitoring systems has had important CV health implications and permits ambulatory CV monitoring on a scale not achievable with traditional cardiac diagnostics.	✓	
19	Heart Rate Variability: An Old Metric with New Meaning in the Era of Using mHealth technologies for Health and Exercise Training Guidance. Part Two: Prognosis and Training [32]	2018	Singh N	USA	Clinical review	Heart rate variability (HRV) is predictive of all-cause and cardiovascular mortality using clinical ECG recordings. the association between HRV and risk stratification is addressed by reviewing the current evidence from data acquired by resting ECG, exercise ECG and medical ambulatory devices.	✓	
20	Atrial fibrillation detection using single lead portable electrocardiographic monitoring: a systematic review and meta-analysis [26]	2018	Ramkumar S	Australia	Systematic review and meta-analysis	Single-lead portable ECG devices may offer an efficient screening option for AF compared with 24hours Holter monitoring. Total monitoring time is related to AF detection and a total of 19 min may achieve a similar detection rate to 24 h Holter monitoring.	✓	
21	The Emerging Role of Wearable Technologies in Detection of Arrhythmia [37]	2018	Cheung CC	Canada	Review	Future wearables will benefit from improved reliability and accuracy, collect additional health and fitness parameters, support management of chronic disease, and provide real-time connectivity and feedback that may supplant conventional medical monitoring.	✓	
22	Wearable Health Devices-Vital Sign Monitoring, Systems and Technologies [11]	2018	Dias D	Portugal	Narrative review	The technology revolution in the miniaturization of electronic devices is enabling to design more reliable and adaptable wearables, contributing for a world-wide change in the health monitoring approach.	✓	
23	Noncontact Wearable Wireless ECG Systems for Long-Term Monitoring [19]	2018	Majumder S	Canada	Review	A wireless ECG monitoring system is developed using flexible and dry capacitive electrodes for long-term monitoring of cardiovascular health.	✓	
24	Breathing Rate Estimation From the Electrocardiogram and Photoplethysmogram: A Review [34]	2018	Charlton PH	UK	Review	Breathing rate (BR) is a key physiological parameter used in a range of clinical settings. A plethora of algorithms have been proposed to estimate BR from the ECG and PPG signals. These BR algorithms provide opportunity for automated, electronic, and unobtrusive measurement of BR in both healthcare and fitness monitoring.	✓	
25	Can Wearable Devices Accurately Measure Heart Rate Variability? A systematic Review [21]	2018	Georgiou K	Greece	Systematic review	The correlation between classic ECG derived HRV and the wearable RV ranged from very good to excellent during rest, yet it declined progressively as exercise level increased.	✓	
26	Portable out-of-hospital electrocardiography: A review of current technologies [18]	2018	Bansal A	India	Narrative review	Number of first-generation devices using a single lead to record cardiac rhythm have been manufactured, tested and are approved by regulatory agencies. These devices are best suited for a short-term rhythm analysis. Second generation devices that can record multiple leads.	✓	
27	Smartphone Apps Using Photoplethysmography for Heart Rate Monitoring: Meta-Analysis [23]	2018	De Ridder B	Belgium	Systematic review and meta-analysis	Heart rate measured by smartphone apps performing PPG agrees with a validated method in an adult population in resting sinus rhythm, provided that during measurement the measuring point was kept still, and that appropriate pressure was maintained. In a pediatric population, the use of these apps can currently not be supported.	✓	
28	From Pacemaker to Wearable: Techniques for ECG Detection Systems [28]	2018	Kumar A	India	Review	Wearable ECG detectors can achieve sensitivity and specificities of around 99% without significant computational efforts, compared to implantable PMs.	✓	✓
29	Wearable Devices in Clinical Trials: Hype and Hypothesis [38]	2018	Izmailova ES	USA	Review	Wearable technologies are promising and have the potential to fundamentally change healthcare by changing the means of collecting, processing, and visualizing health data along with a reduction of healthcare costs. Remote data collection can bring new treatments and care management to all patients in need.	✓	
30	Smart health and innovation: facilitating health-related behaviour change [16]	2017	Redfern J	Australia	Narrative review	Smart health and innovation are evolving rapidly and may help with diagnosis, treatment, and management of chronic diseases including a focus on nutrition and its role in health.	✓	
31	ECG by mobile technologies [39]	2016	Guzik P	Poland	Narrative review	At present, the use of mobile ECG technology has not reached recognized guidelines and/or accepted clinical recommendations. The solid clinical evidence showing the real usefulness of new mobile ECG technologies should be available soon from large, randomized, multisite and prospective studies.	✓	
32	mHealth in Cardiovascular Health Care [15]	2016	Chow CK	Australia	Narrative review	mHealth has the potential to reduce socio economic disparity and alleviate the burden of CV disease. It includes simple strategies such the use of SMS for smoking-cessation, weight loss and diabetes management programmes. mHealth can also involve complex strategies such apps, GPS and Bluetooth technologies.	✓	
33	Use of smartphone technology in cardiology [35]	2016	Nguyen HH	USA	Review	The ever-broadening connectivity and increasing capabilities of smartphone-based technologies can better monitor, diagnose, and prevent cardiovascular diseases. Researchers can leverage the ubiquitous use of smartphone-based technologies and their constant stream of biometric data to establish large community based clinical research studies.	✓	
34	Electrocardiographic patch devices and contemporary wireless cardiac monitoring [20]	2015	Fung E	USA	Narrative review	Where continuous ECG monitoring in the short to medium term (days to weeks) is indicated, these cardiac devices and related digital mobile health technologies are reshaping the clinician-patient interface with important implications for future health care delivery.	✓	✓

## 5. Conclusions

According to a 2017 report by the International Labour Office (ILO), around 150 workers have an accident at work every 15 s, with a global cost due to non-fatal injuries of over USD 300 million. Accidents at work remain a huge inter-industrial problem, despite the increasingly strict safety rules and procedures with more than 300,000 workers deaths per year. The ILO itself identifies circulatory diseases as the largest killer on a global level, as was also reported by the World Health Organization (WHO), with the highest impact in terms of DALYs (years of life lost or years lived with disabilities).

This narrative review aims to summarise advancements in the development of wearable and smart devices for cardiovascular monitoring and their possible applications, demonstrating the positive trends associated to using and deploying mobile ECG devices across different environmental settings and populations. More recent models are also equipped with built-in electrodes for measuring cardiac activity, according to what Einthoven did just over a century ago, thanks to a closed circuit between the heart and both arms. These devices, through interfacing with other devices, such as smartphones, allow for real-time ECG recording, the storing of such data and, if necessary, its electronic transmission.

The occupational physician, in the industry 4.0 scenario, characterized by an incessant technological progress, attributable to what was envisaged by Moore in the mid-1960s, and perhaps well beyond, as well as emerging risks, is expected to play a pivotal role in the H&S process, also being required to evaluate aspects of ethical nature related to the use of smart devices, such as the management of collected data related to physiological parameters and the location of the worker. We firmly believe that technology, if applied in compliance with the necessary ethical principles, can contribute to improving the health and safety of workers. It will fill some lacunae, such as for lone working and return to work issues, with the consequent further reduction of occupational risks. Last but not least, this technology is to be considered as having the potential to assess physiological parameters and monitoring campaigns. It is not aimed towards the diagnosis of any pathological conditions, which should always be on charge of the medical specialist.

More research is needed so as to clarify the use of these wearable and smart devices in the area of occupational medicine. These studies will have to consider the differences in health disparities, health literacy and technology access, influenced by demographic factors, such as socioeconomic status, rural versus urban living situation, gender, age, race and ethnicity. Finally, further studies are needed to validate the actual reliability of these devices, both in the medical and preventive field.

## References

[1] Fye W.B. (1994). A History of the origin, evolution, and impact of electrocardiography. Am. J. Cardiol..

[2] Henson J.R. (1971). Descartes and the ECG lettering series. J. Hist. Med. Allied Sci..

[3] Pahlm O., Uvelius B. (2019). The winner takes it all: Willem Einthoven, Thomas Lewis, and the Nobel prize 1924 for the discovery of the electrocardiogram. J. Electrocardiol..

[4] AlGhatrif M., Lindsay J. (2012). A brief review: History to understand fundamentals of electrocardiography. J. Community Hosp. Intern. Med. Perspect..

[5] Rivera-Ruiz M., Cajavilca C., Varon J. (2008). Einthoven’s string galvanometer: The first electrocardiograph. Tex. Heart Inst. J..

[6] Yamagishi T. (2011). A Short Biography of Takemi Taro, the President of the Japan Medical Association. J. Nanzan Acad. Soc. Soc. Sci..

[7] Yang X.-L., Liu G.-Z., Tong Y.-H., Yan H., Xu Z., Chen Q., Liu X., Zhang H.-H., Wang H.-B., Tan S.-H. (2015). The history, hotspots, and trends of electrocardiogram. J. Geriatr. Cardiol..

[8] Baule G.M., McFee R. (1963). Detection of the magnetic field of the heart. Am. Heart J..

[9] Miller S.A., Forrest J.L. (2001). Enhancing your practice through evidence-based decision making: PICO, learning how to ask good questions. J. Evid. Based Dent. Pract..

[10] Booth A. (2016). Clear and present questions: Formulating questions for evidence-based practice. Libr. Hi. Tech..

[11] Dias D., Paulo Silva Cunha J. (2018). Wearable Health Devices - Vital Sign Monitoring, Systems and Technologies. Sensors.

[12] Huang H., Su S., Wu N., Wan H., Wan S., Bi H., Sun L. (2019). Graphene-Based Sensors for Human Health Monitoring. Front. Chem..

[13] Soroudi A., Hernández N., Berglin L., Nierstrasz V. (2019). Electrode placement in electrocardiography smart garments: A review. J. Electrocardiol..

[14] Teferra M.N., Kourbelis C., Newman P., Ramos J.S., Hobbs D., Clark R.A., Reynolds K.J. (2019). Electronic textile electrocardiogram monitoring in cardiac patients. JBI Database Syst. Rev. Implement. Rep..

[15] Chow C.K., Ariyarathna N., Islam S.M., Thiagalingam A., Redfern J. (2016). mHealth in Cardiovascular Health Care. Heart Lung Circ..

[16] Redfern J. (2017). Smart health and innovation: Facilitating health-related behavior change. Proc. Nutr. Soc..

[17] Duarte R., Stainthorpe A., Mahon J., Greenhalgh J., Richardson M., Nevitt S., Kotas E., Boland A., Thom H., Marshall T. (2019). Lead-I ECG for detecting atrial fibrillation in patients attending primary care with an irregular pulse using single-time point testing: A systematic review and economic evaluation. PLoS ONE.

[18] Bansal A., Joshi R. (2018). Portable out-of-hospital electrocardiography: A review of current technologies. J. Arrhythm..

[19] Majumder S., Chen L., Marinov O., Chen C.H., Mondal T., Deen M.J. (2018). Noncontact Wearable Wireless ECG Systems for Long-Term Monitoring. IEEE Rev. Biomed. Eng..

[20] Fung E., Jarvelin M., Doshi R.N., Shinbane J.S., Carlson S.K., Grazette L.P., Chang P.M., Sangha R.S., Huikuri H.V., Peters N.S. (2015). Electrocardiographic patch devices and contemporary wireless cardiac monitoring. Front. Physiol..

[21] Georgiou K., Larentzakis A.V., Khamis N.N., Alsuhaibani G.I., Alaska Y.A., Giallafos E.J. (2018). Can Wearable Devices Accurately Measure Heart Rate Variability? A Systematic Review. Folia Med. (Plovdiv).

[22] Sajeev J.K., Koshy A.N., Teh A.W. (2019). Wearable devices for cardiac arrhythmia detection: A new contender?. Intern. Med. J..

[23] De Ridder B., Van Rompaey B., Kampen J.K., Haine S., Dilles T. (2018). Smartphone Apps Using Photoplethysmography for Heart Rate Monitoring: Meta-Analysis. JMIR Cardio..

[24] Sanders D., Ungar L., Eskander M.A., Seto A.H. (2019). Ambulatory ECG monitoring in the age of smartphones. Cleve Clin. J. Med..

[25] Raja J.M., Elsakr C., Roman S., Cave B., Pour-Ghaz I., Nanda A., Maturana M., Khouzam R.N. (2019). Apple Watch, Wearables, and Heart Rhythm: Where do we stand?. Ann. Transl. Med..

[26] Ramkumar S., Nerlekar N., D’Souza D., Pol D.J., Kalman J.M., Marwick T.H. (2018). Atrial fibrillation detection using single lead portable electrocardiographic monitoring: A systematic review and meta-analysis. BMJ Open.

[27] Dobbs W.C., Fedewa M.V., Macdonald H.V., Holmes C.J., Cicone Z.S., Plews D.J., Esco M.R. (2019). The Accuracy of Acquiring Heart Rate Variability from Portable Devices: A Systematic Review and Meta-Analysis. Sports Med..

[28] Kumar A., Komaragiri R., Kumar M. (2018). From Pacemaker to Wearable: Techniques for ECG Detection Systems. J. Med. Syst..

[29] Karmen C.L., Reisfeld M.A., McIntyre M.K., Timmermans R., Frishman W. (2019). The Clinical Value of Heart Rate Monitoring Using an Apple Watch. Cardiol. Rev..

[30] Isakadze N., Martin S.S. (2019). How useful is the smartwatch ECG?. Trends Cardiovasc. Med..

[31] Singh N., Moneghetti K.J., Christle J.W., Hadley D., Plews D., Froelicher V. (2018). Heart Rate Variability: An Old Metric with New Meaning in the Era of using mHealth Technologies for Health and Exercise Training Guidance. Part One: Physiology and Methods. Arrhythm. Electrophysiol. Rev..

[32] Singh N., Moneghetti K.J., Christle J.W., Hadley D., Froelicher V., Plews D. (2018). Heart Rate Variability: An Old Metric with New Meaning in the Era of Using mHealth technologies for Health and Exercise Training Guidance. Part Two: Prognosis and Training. Arrhythm. Electrophysiol. Rev..

[33] Elgendi M., Fletcher R., Liang Y., Howard N., Lovell N.H., Abbott D., Lim K., Ward R. (2019). The use of photoplethysmography for assessing hypertension. NPJ Digit Med..

[34] Charlton P.H., Birrenkott D.A., Bonnici T., Pimentel M.A., Johnson A.E.W., Alastruey J., Tarassenko L., Watkinson P., Beale R., Clifton D. (2018). Breathing Rate Estimation From the Electrocardiogram and Photoplethysmogram: A Review. IEEE Rev. Biomed. Eng..

[35] Nguyen H.H., Silva J.N. (2016). Use of smartphone technology in cardiology. Trends Cardiovasc. Med..

[36] Alharbi M., Straiton N., Smith S., Neubeck L., Gallagher R. (2019). Data management and wearables in older adults: A systematic review. Maturitas.

[37] Cheung C.C., Krahn A.D., Andrade J.G. (2018). The Emerging Role of Wearable Technologies in Detection of Arrhythmia. Can. J. Cardiol..

[38] Izmailova E.S., Wagner J.A., Perakslis E.D. (2018). Wearable Devices in Clinical Trials: Hype and Hypothesis. Clin. Pharmacol. Ther..

[39] Guzik P., Malik M. (2016). ECG by mobile technologies. J. Electrocardiol..

[40] Jo A., Coronel B.D., Coakes C.E., Mainous A.G. (2019). Is There a Benefit to Patients Using Wearable Devices Such as Fitbit or Health Apps on Mobiles? A Systematic Review. Am. J. Med..

[41] Al-Alusi M.A., Ding E., McManus D.D., Lubitz S.A. (2019). Wearing Your Heart on Your Sleeve: The Future of Cardiac Rhythm Monitoring. Curr. Cardiol. Rep..

[42] Solbiati M., Trombetta L., Sacco R., Erba L., Bozzano V., Costantino G., Raj S.R., Barbic F., Casazza G., DiPaola F. (2019). A Systematic Review of Noninvasive Electrocardiogram Monitoring Devices for the Evaluation of Suspected Cardiovascular Syncope. J. Med. Devices..

[43] Jacobs J.V., Hettinger L.J., Huang Y.H., Jeffries S., Lesch M.F., Simmons L.A., Verma S.K., Willetts J.L. (2019). Employee acceptance of wearable technology in the workplace. Appl. Ergon..

[44] Maltseva K. (2020). Wearables in the workplace: The brave new world of employee engagement. Bus. Horiz..

[45] Li C.K., White F.A., Tipoe T., Liu T., Wong M.C., Jesuthasan A., Baranchuk A., Tse G., Yan B.P., Borges L. (2019). The Current State of Mobile Phone Apps for Monitoring Heart Rate, Heart Rate Variability, and Atrial Fibrillation: Narrative Review. JMIR Mhealth Uhealth.

[46] Marston H.R., Hadley R., Banks D., Duro M.D.C.M. (2019). Mobile Self-Monitoring ECG Devices to Diagnose Arrhythmia that Coincide with Palpitations: A Scoping Review. Healthcare (Basel).

